# Supplementary material for: Contribution of genetic factors to high rates of neonatal hyperbilirubinaemia on the Thailand-Myanmar border
Source: PLOS Glob Public Health. 2022 Jun 17;2(6):e0000475. doi: 10.1371/journal.pgph.0000475 (PMC10021142; doi:10.1371/journal.pgph.0000475)
Supplement: S1 Table — (DOCX) [file pgph.0000475.s001.docx]

**Contribution of genetic factors to high rates of neonatal hyperbilirubinaemia on the Thailand-Myanmar border**

**S1 Table.** Primers, PCR conditions, restriction enzyme and band interpretation for genotyping of allele *6 and promoter of the human UGT1A1 gene.

| **Assay** | **Oligonucleotide sequence** | **Annealing temperature** | **Restriction enzyme** | **Fragments’ size** |
| --- | --- | --- | --- | --- |
| UGT1A1*6 | 5’-AGATACTGTTGATCCCAGTG-3’  5’-CTTCAAGGTGTAAAATGGTC-3’ | 56°C | *AvaII* | WT=146bp  Mutation=126bp |
| UGT1A1 Promoter | 5’-TAA CTT GGT GTA TCG ATT GGT-3’  5’-CTT TGC TCC TGC CAG AGG TT-3’ | 58°C | N.A | TA5=71bp (*36) TA6=73bp (WT)  TA7=75bp (*28)  TA8=77bp (*37) |
