# Supplementary material for: Contribution of genetic factors to high rates of neonatal hyperbilirubinaemia on the Thailand-Myanmar border
Source: PLOS Glob Public Health. 2022 Jun 17;2(6):e0000475. doi: 10.1371/journal.pgph.0000475 (PMC10021142; doi:10.1371/journal.pgph.0000475)
Supplement: S2 Table — (DOCX) [file pgph.0000475.s002.docx]

**Contribution of genetic factors to high rates of neonatal hyperbilirubinaemia on the Thailand-Myanmar border**

**S2 Table**. Uni- and multivariable analysis of potential risk factors and genotyping for developing NH in the first week of life using mixed effect Cox proportional hazard model clustering by site

| Characteristics | Neonates with NH, n (%)  (N=309) | Neonates without NH, n (%)  (N=961) | Univariable analysis | | Multivariable analysis*^a^* | |
| --- | --- | --- | --- | --- | --- | --- |
|  |  |  | HR (95% CI) | p-value | HR (95% CI) | p-value |
| Newborn genotyping |  |  |  |  |  |  |
| G6PD (any mutation) | (N=296) | (N=929) |  |  |  |  |
| WT | 205 (69) | 801 (86) | Reference |  | Reference |  |
| Heterozygote | 40 (14) | 83 (9) | 1.76 (1.25, 2.47) | 0.001 | 2.09 (1.41, 3.12) | <0.001 |
| Hemi + Homozygote | 51 (17) | 45 (5) | 3.58 (2.63, 4.86) | <0.001 | 4.78 (3.35, 6.84) | <0.001 |
| UGT1A1*6 | (N=297) | (N=928) |  |  |  |  |
| WT | 188 (63) | 657 (71) | Reference |  | Reference |  |
| Heterozygote | 90 (30) | 253 (27) | 1.18 (0.91, 1.51) | 0.206 | 1.24 (0.92, 1.66) | 0.151 |
| Homozygote | 19 (6) | 18 (2) | 2.50 (1.55, 4.01) | <0.001 | 3.22 (1.94, 5.37) | <0.001 |
| UGT1A1*28 | (N=244) | (N=847) |  |  |  |  |
| WT (TA6/6) | 204 (84) | 646 (76) | Reference |  | Reference |  |
| Hetero and homozygote (TA6/7+TA7/7) | 40 (16) | 201 (24) | 0.67 (0.48, 0.95) | 0.022 | 0.77 (0.53, 1.11) | 0.160 |
| Maternal Characteristics |  |  |  |  |  |  |
| Young maternal age (≤20 y, min=15) | 103 (33) | 254 (26) | 1.39 (1.10, 1.76) | 0.007 | * | * |
| Illiterate (cannot read) | 102 (33) | 346 (36) | 0.88 (0.69, 1.11) | 0.275 |  |  |
| Smoking | 30 (10) | 85 (9) | 1.01 (0.69, 1.48) | 0.953 |  |  |
| Primigravida (Primipara) | 139 (45) | 296 (31) | 1.81 (1.45, 2.27) | <0.001 | 1.71 (1.30, 2.25) | <0.001 |
| Overweight | 74/299 (25) | 223/952 (23) | 1.05 (0.81, 1.36) | 0.707 |  |  |
| Pre-eclampsia or eclampsia | 14 (5) | 15 (2) | 2.59 (1.51, 4.43) | <0.001 | 1.60 (0.84, 3.04) | 0.155 |
| Haemoglobinopathies | 24 (8) | 67 (7) | 1.00 (0.66, 1.51) | 0.982 |  |  |
| Obstetric characteristics |  |  |  |  |  |  |
| Rupture of membranes ≥ 18h | 30/297 (10) | 52/948 (5) | 1.76 (1.21, 2.57) | 0.003 | 2.30 (1.50, 3.53) | <0.001 |
| Oxytocin infusion | 35/308 (11) | 92 (10) | 1.22 (0.86, 1.73) | 0.276 |  |  |
| Delayed cord clamping | 251 (81) | 853 (89) | 0.59 (0.44, 0.78) | <0.001 | 0.78 (0.54, 1.13) | 0.188 |
| Neonatal Characteristics |  |  |  |  |  |  |
| Gestational age (<38 weeks) | 120 (39) | 26 (3) | 12.6 (10.0, 15.8) | <0.001 | 15.0 (11.3, 20.0) | <0.001 |
| Resuscitation | 13/308 (4) | 26/960 (3) | 1.38 (0.79, 2.40) | 0.260 |  |  |
| Presence of haematoma | 22 (7) | 29/ 960 (3) | 2.24 (1.45, 3.46) | <0.001 | 1.98 (1.18, 3.32) | 0.010 |
| Sgaw Karen ethnicity | 153/301 (51) | 350/936 (37) | 1.36 (1.04, 1.77) | 0.025 | 1.29 (0.95, 1.74) | 0.104 |
| Male sex | 179 (58) | 481 (50) | 1.32 (1.06, 1.66) | 0.015 | * | * |
| Small for gestational age | 54 (17) | 195 (20) | 0.86 (0.64, 1.15) | 0.305 |  |  |
| Sibling with history of jaundice | 37 (12) | 97 (10) | 1.04 (0.74, 1.48) | 0.809 |  |  |
| Use of naphthalene for storing the clothes | 16 (5) | 50 (5) | 1.01 (0.61, 1.68) | 0.954 |  |  |
| G6PD deficiency (by FST) | 49 (16) | 41 (4) | 3.33 (2.46, 4.53) | <0.001 | * | * |
| Potential ABO incompatibility | 54 (17) | 135 (14) | 1.32 (0.98, 1.76) | 0.068 | 1.30 (0.93, 1.82) | 0.131 |
| Positive Coombs test | 13/290 (4) | 31/934 (3) | 1.32 (0.76, 2.31) | 0.322 |  |  |
| Clinical events |  |  |  |  |  |  |
| Severe infection 0-24h | 24 (8) | 39 (4) | 1.93 (1.27, 2.92) | 0.002 | 1.62 (1.00, 2.64) | 0.052 |
| Weight loss ≥7% at 24h [12-30h] of life | 11 (4) | 23 (2) | 1.44 (0.79, 2.62) | 0.239 |  |  |
| HCT at at 24 [12-30h] of life | (N=307)  mean (SD)  59.5 (7.6) | (N=954)  mean (SD)  59.1 (7.1) | 1.11 (0.95, 1.30)  (Per 10-unit increment) | 0.195 |  |  |
| Polycythaemia (HCT >70%) at 24 [12-30h] of life | 31 (10) | 81 (8) | 1.27 (0.88, 1.85) | 0.204 |  |  |

*WT: wild type; HR: Hazard ratio; CI: confidence interval.*

*^a^ Adjusted for Primigravida, Pre-eclampsia or eclampsia, Rupture of membrane ≥ 18h, Delayed cord clamping, Gestational age <38 weeks, Presence of hematoma, Sgaw Karen ethnicity, Potential ABO incompatibility, Severe infection 0-24h and genotyping of G6PD, UGT1A1*6 and UGT1A1*28 with p<0.15 from univariate model. There was no interaction effect between G6PD status (Hemi-Homo/Heterozygote vs WT) and UGT1A1*6 status (homozygote vs non- homozygote), p=0.171. Harrell’s C statistic for model discrimination = 0.818.*

** Young maternal age, Oxytocin infusion, and Gender and G6PD deficiency were significant in univariable model but not be included in the multivariable model because they were highly correlated with Primigravida, Rupture of membrane ≥ 18h, and G6PD genotyping, respectively.*
