# Supplementary material for: Contribution of genetic factors to high rates of neonatal hyperbilirubinaemia on the Thailand-Myanmar border
Source: PLOS Glob Public Health. 2022 Jun 17;2(6):e0000475. doi: 10.1371/journal.pgph.0000475 (PMC10021142; doi:10.1371/journal.pgph.0000475)
Supplement: S3 Table — (DOCX) [file pgph.0000475.s003.docx]

**Contribution of genetic factors to high rates of neonatal hyperbilirubinaemia on the Thailand-Myanmar border**

**S3 Table**. Characteristics of neonates with EGA ≥ 38 weeks who developed NH within 48 hours, between 49 and 168 hours and not develop NH in the first week of life

| Characteristics | Neonates with early NH within 48h,  n (%)  (N=61) | Neonates with late NH 49-168h,  n (%)  (N=128) | Neonates without NH within 168h,  n (%)  (N=935) |
| --- | --- | --- | --- |
| Newborn genotyping |  |  |  |
| G6PD (any mutation) | (N=59) | (N=122) | (N=906) |
| WT | 33 (56) | 83 (68) | 779 (86) |
| Heterozygote | 8 (14) | 19 (16) | 82 (9) |
| Hemi + Homozygote | 18 (31) | 20 (16) | 45 (5) |
| UGT1A1*6 | (N=60) | (N=122) | (N=905) |
| WT | 42 (70) | 67 (55) | 639 (71) |
| Heterozygote | 15 (25) | 42 (34) | 248 (27) |
| Homozygote | 3 (5) | 13 (11) | 18 (2) |
| UGT1A1*28 | (N=52) | (N=110) | (N=827) |
| WT (TA6/6) | 38 (73) | 100 (91) | 629 (76) |
| Hetero and homozygote (TA6/7+ TA7/7) | 14 (27) | 10 (9) | 198 (24) |
| Maternal Characteristics |  |  |  |
| Young maternal age (≤20 y, min=15) | 12 (20) | 40 (31) | 246 (26) |
| Illiterate (cannot read) | 23 (38) | 37 (29) | 333 (36) |
| Smoking | 4 (7) | 13 (10) | 80 (9) |
| Primigravida (Primipara) | 27 (44) | 55 (43) | 291 (31) |
| Overweight | 25/59 (42) | 34 (27) | 228/926 (24) |
| Pre-eclampsia or eclampsia | 5 (8) | 2 (2) | 14 (2) |
| Haemoglobinopathies | 2/60 (3) | 18/126 (14) | 66/923 (7) |
| Obstetric characteristics |  |  |  |
| Rupture of membranes ≥ 18h | 6/60 (10) | 12/124 (10) | 52/923 (6) |
| Oxytocin infusion | 16 (26) | 15 (12) | 92 (10) |
| Delayed cord clamping | 45 (74) | 109 (85) | 831 (89) |
| Neonatal Characteristics |  |  |  |
| Resuscitation | 4 (7) | 7 (5) | 26/934 (3) |
| Presence of haematoma | 7 (11) | 11 (9) | 29/ 934 (3) |
| Sgaw Karen ethnicity | 22/59 (37) | 73/124 (59) | 341/910 (37) |
| Male sex | 36 (59) | 75 (59) | 472 (50) |
| Small for gestational age | 11 (18) | 24 (19) | 185 (20) |
| Sibling with history of jaundice | 10 (16) | 17 (13) | 93 (10) |
| Use of naphthalene for storing the clothes | 3 (5) | 8 (6) | 48 (5) |
| G6PD deficiency (by FST) | 18 (30) | 19 (15) | 41 (4) |
| Potential ABO incompatibility | 14 (23) | 20 (16) | 132 (14) |
| Positive Coombs test | 5/58 (9) | 3/123 (2) | 29/909 (3) |
| Clinical events |  |  |  |
| Severe infection 0-24h | 2 (3) | 10 (8) | 37 (4) |
| Weight loss ≥7% at 24h [12-30h] of life | 2 (3) | 4 (3) | 23 (2) |
| HCT at 24h [12-30h] of life | mean (SD)  59.5 (8.3) | mean (SD), (N=62)  59.1 (7.1) | mean (SD), (N=928)  59.1 (7.1) |
| Polycythaemia (HCT >70%) at 24 [12-30h] of life | 8 (13) | 11 (9) | 79 ( 8) |
