# Supplementary material for: Contribution of genetic factors to high rates of neonatal hyperbilirubinaemia on the Thailand-Myanmar border
Source: PLOS Glob Public Health. 2022 Jun 17;2(6):e0000475. doi: 10.1371/journal.pgph.0000475 (PMC10021142; doi:10.1371/journal.pgph.0000475)
Supplement: S4 Table — (DOCX) [file pgph.0000475.s004.docx]

**Contribution of genetic factors to high rates of neonatal hyperbilirubinaemia on the Thailand-Myanmar border**

**S4 Table.** Early NH: Uni- and multivariable analysis of potential risk factors using a mixed effects Cox proportional hazard model clustering by site among neonates ≥ 38 weeks who developed NH early (within 48 hours) and neonates who did not develop NH in the first week of life.

| Characteristics | Univariable analysis | | Multivariable analysis^a^ | |
| --- | --- | --- | --- | --- |
|  | HR (95% CI) | p-value | HR (95% CI) | p-value |
| Newborn genotyping |  |  |  |  |
| G6PD (any mutation) |  |  |  |  |
| WT | Reference |  | Reference |  |
| Heterozygote | 2.27 (1.05, 4.91) | 0.038 | 2.61 (1.18, 5.77) | 0.018 |
| Hemi + Homozygote | 8.75 (4.93, 15.54) | <0.001 | 9.18 (4.79, 17.59) | <0.001 |
| UGT1A1*6 |  |  |  |  |
| WT | Reference |  | Reference |  |
| Heterozygote | 0.92 (0.51, 1.66) | 0.781 | 1.17 (0.64, 2.17) | 0.608 |
| Homozygote | 2.49 (0.77, 8.03) | 0.127 | 1.47 (0.43, 5.10) | 0.540 |
| UGT1A1*28 |  |  |  |  |
| WT (TA6/6) | Reference |  |  |  |
| Hetero and homozygote ( TA6/7+ TA7/7) | 1.17 (0.63, 2.16) | 0.620 |  |  |
| Maternal Characteristics |  |  |  |  |
| Young maternal age (≤20 y) | 0.69 (0.37, 1.30) | 0.248 |  |  |
| Illiterate (cannot read) | 1.09 (0.65, 1.83) | 0.737 |  |  |
| Smoking | 0.75 (0.27, 2.08) | 0.586 |  |  |
| Primigravida (Primipara) | 1.74 (1.05, 2.88) | 0.032 | 2.06 (1.10, 3.88) | 0.024 |
| Overweight | 2.35 (1.41, 3.95) | 0.001 | 2.15 (1.20, 3.88) | 0.011 |
| Pre-eclampsia or eclampsia | 5.43 (2.18, 13.56) | <0.001 | 1.69 (0.98, 2.93) | 0.060 |
| Haemoglobinopathies | 0.45 (0.11, 1.85) | 0.269 |  |  |
| Obstetric characteristics |  |  |  |  |
| Rupture of membranes ≥ 18h | 1.83 (0.79, 4.26) | 0.159 |  |  |
| Oxytocin infusion | 3.16 (1.79, 5.59) | <0.001 | 1.18 (0.51, 2.70) | 0.696 |
| Delayed cord clamping | 0.36 (0.20, 0.64) | <0.001 | 0.38 (0.17, 0.84) | 0.018 |
| Neonatal Characteristics |  |  |  |  |
| Resuscitation | 2.40 (0.87, 6.61) | 0.091 | 0.78 (0.24, 2.61) | 0.691 |
| Presence of haematoma | 3.85 (1.75, 8.47) | 0.001 | 1.66 (0.63, 4.36) | 0.081 |
| Sgaw Karen ethnicity | 0.99 (0.59, 1.68) | 0.975 |  |  |
| Male sex | 1.41 (0.84, 2.34) | 0.190 |  |  |
| Small for gestational age | 0.90 (0.47, 1.72) | 0.741 |  |  |
| Sibling with history of jaundice | 1.75 (0.89, 3.45) | 0.104 | 2.08 (0.88, 4.90) | 0.094 |
| Use of naphthalene for storing the clothes | 0.96 (0.30, 3.05) | 0.941 |  |  |
| G6PD deficiency (by FST) | 8.39 (4.84, 14.54) | <0.001 |  |  |
| Potential ABO incompatibility | 1.80 (0.99, 3.26) | 0.054 | 1.69 (0.82, 3.51) | 0.156 |
| Positive Coombs test | 2.81 (1.12, 7.02) | 0.027 | 1.41 (0.46, 4.36) | 0.551 |
| Clinical events |  |  |  |  |
| Severe infection 0-24h | 0.82 (0.20, 3.37) | 0.786 |  |  |
| Weight loss ≥7% at 24h [12-30h] of life | 1.33 (0.33, 5.46) | 0.689 |  |  |
| HCT at 24h [12-30h] of life | 1.10 (0.77, 1.56)  (per 10-uint increment) | 0.607 |  |  |
| Polycythaemia (HCT >70%) at 24 [12-30h] of life | 1.63 (0.78, 3.43) | 0.197 |  |  |

*WT: wild type; HR: Hazard ratio; CI: confidence interval*

*^a^Adjusted for Primigravida, Overweight, Pre-eclampsia or eclampsia, Oxytocin infusion, Delayed cord clamping, Resuscitation, Presence of hematoma, Sibling with history of jaundice, Potential ABO incompatibility, Positive Coombs test and genotyping of G6PD and UGT1A1*6 with p<0.15 from univariate model. Sex and G6PD deficiency by FST were significant in univariable model but not be included in the* *multivariable model because they were highly correlated with G6PD genotyping. Harrell’s C statistic for model discrimination = 0.824.*
