# Supplementary material for: Contribution of genetic factors to high rates of neonatal hyperbilirubinaemia on the Thailand-Myanmar border
Source: PLOS Glob Public Health. 2022 Jun 17;2(6):e0000475. doi: 10.1371/journal.pgph.0000475 (PMC10021142; doi:10.1371/journal.pgph.0000475)
Supplement: S5 Table — (DOCX) [file pgph.0000475.s005.docx]

**Contribution of genetic factors to high rates of neonatal hyperbilirubinaemia on the Thailand-Myanmar border**

**S5 Table.** Late NH: Uni- and multivariable analysis of potential risk using mixed effects Cox proportional hazard model clustering by site among neonates ≥ 38 weeks who developed NH late (49-168 hours) and neonates who did not develop NH in the first week of life.

| Characteristics | Univariable analysis | | Multivariable analysis^a^ | |
| --- | --- | --- | --- | --- |
|  | HR (95% CI) | p-value | HR (95% CI) | p-value |
| Newborn genotyping |  |  |  |  |
| G6PD (any mutation) |  |  |  |  |
| WT | Reference |  | Reference |  |
| Heterozygote | 2.07 (1.26, 3.41) | 0.004 | 2.16 (1.23, 3.78) | 0.007 |
| Hemi + Homozygote | 3.59 (2.20, 5.85) | <0.001 | 4.40 (2.51, 7.71) | <0.001 |
| UGT1A1*6 |  |  |  |  |
| WT | Reference |  | Reference |  |
| Heterozygote | 1.51 (1.03, 2.23) | 0.036 | 1.34 (0.87, 2.06) | 0.179 |
| Homozygote | 4.46 (2.45, 8.11) | <0.001 | 3.77 (2.01, 7.08) | <0.001 |
| UGT1A1*28 |  |  |  |  |
| WT (TA6/6) | Reference |  | Reference |  |
| Hetero and homozygote ( TA6/7+ TA7/7) | 0.34 (0.18, 0.65) | 0.001 | 0.40 (0.20, 0.81) | 0.011 |
| Maternal Characteristics |  |  |  |  |
| Young maternal age (≤20 y) | 1.27 (0.87, 1.85) | 0.209 |  |  |
| Illiterate (cannot read) | 0.74 (0.50, 1.08) | 0.118 | 0.77 (0.49, 1.22) | 0.268 |
| Smoking | 1.03 (0.58, 1.84) | 0.913 |  |  |
| Primigravida (Primipara) | 1.72 (1.21, 2.44) | 0.002 | 1.51 (0.99, 2.29) | 0.056 |
| Overweight | 1.16 (0.78, 1.72) | 0.454 |  |  |
| Pre-eclampsia or eclampsia | 0.98 (0.24, 3.96) | 0.975 |  |  |
| Haemoglobinopathies | 1.81 (1.10, 2.99) | 0.020 | 1.88 (1.09, 3.26) | 0.024 |
| Obstetric characteristics |  |  |  |  |
| Rupture of membranes ≥ 18h | 1.63 (0.90, 2.97) | 0.107 | 2.02 (1.07, 3.82) | 0.030 |
| Oxytocin infusion | 1.25 (0.73, 2.14) | 0.418 |  |  |
| Delayed cord clamping | 0.74 (0.45, 1.20) | 0.217 |  |  |
| Neonatal Characteristics |  |  |  |  |
| Resuscitation | 1.68 (0.78, 3.62) | 0.182 |  |  |
| Presence of haematoma | 2.81 (1.52, 5.22) | 0.001 | 2.04 (1.01, 4.11) | 0.047 |
| Sgaw Karen ethnicity | 1.80 (1.17, 2.77) | 0.007 | 1.51 (0.92, 2.49) | 0.103 |
| Male sex | 1.34 (0.94, 1.90) | 0.103 |  |  |
| Small for gestational age | 0.95 (0.61, 1.49) | 0.834 |  |  |
| Sibling with history of jaundice | 1.13 (0.67, 1.89) | 0.643 |  |  |
| Use of naphthalene for storing the clothes | 1.22 (0.60, 2.50) | 0.588 |  |  |
| G6PD deficiency (by FST) | 3.21 (1.97, 5.22) | <0.001 |  |  |
| Potential ABO incompatibility | 1.19 (0.74, 1.91) | 0.486 |  |  |
| Positive Coombs test | 0.73 (0.23, 2.30) | 0.591 |  |  |
| Clinical events |  |  |  |  |
| Severe infection 0-24h | 2.05 (1.08, 3.92) | 0.029 | 2.29 (1.12, 4.67) | 0.023 |
| Weight loss ≥7% at 24h [12-30h] of life | 1.26 (0.47, 3.42) | 0.647 |  |  |
| HCT at 24h [12-30h] of life | 1.14 (0.89, 1.47)  (per 10-unit increment) | 0.304 |  |  |
| Polycythaemia (HCT >70%) at 24 [12-30h] of life | 1.12 (0.60, 2.08) | 0.726 |  |  |

*WT: wild type; HR: Hazard ratio; CI: confidence interval.*

*^a^Adjusted for Literacy****,*** *Primigravida, Rupture of membrane ≥ 18h, Presence of hematoma, Sgaw Karen ethnicity, Severe infection 0-24h and genotyping of G6PD, UGT1A1*6, UGT1A1 promoter and mother’s haemoglobinopathies* *with p<0.15 from univariate model. Sex and G6PD deficiency by FST were* *significant in univariable model but not be included in the multivariable model because they were highly correlated with G6PD genotyping, N=886. Harrell’s C statistic for model discrimination = 0.753.*
