# Supplementary material for: Contribution of genetic factors to high rates of neonatal hyperbilirubinaemia on the Thailand-Myanmar border
Source: PLOS Glob Public Health. 2022 Jun 17;2(6):e0000475. doi: 10.1371/journal.pgph.0000475 (PMC10021142; doi:10.1371/journal.pgph.0000475)
Supplement: S6 Table — (DOCX) [file pgph.0000475.s006.docx]

**Contribution of genetic factors to high rates of neonatal hyperbilirubinaemia on the Thailand-Myanmar border**

**S6 Table.**  Uni- and multivariable analysis of potential risk factors and genotyping for developing NH in the first week of life using mixed effects Cox proportional hazard model clustering by site among neonates who develop NH after 72 hours of life and neonates who did not develop NH in the first week of life.

| Characteristics | Univariable analysis | | Multivariable analysis^a^ | |
| --- | --- | --- | --- | --- |
|  | HR (95% CI) | p-value | HR (95% CI) | p-value |
| Newborn genotyping |  |  |  |  |
| G6PD (any mutation) |  |  |  |  |
| WT | Reference |  | Reference |  |
| Heterozygote | 1.95 (0.95, 4.00) | 0.069 | 2.26 (1.02, 5.02) | 0.046 |
| Hemi + Homozygote | 2.93 (1.38, 6.22) | 0.005 | 3.45 (1.41, 8.46) | 0.007 |
| UGT1A1*6 |  |  |  |  |
| WT | Reference |  | Reference |  |
| Heterozygote | 1.54 (0.87, 2.74) | 0.139 | 1.18 (0.61, 2.30) | 0.625 |
| Homozygote | 8.32 (4.14, 16.70) | <0.001 | 7.36 (3.48, 15.59) | <0.001 |
| UGT1A1*28 |  |  |  |  |
| WT (TA6/6) | Reference |  | Reference |  |
| Hetero and homozygote ( TA6/7+ TA7/7) | 0.25 (0.09, 0.71) | 0.008 | 0.29 (0.09, 0.98) | 0.046 |
| Maternal Characteristics |  |  |  |  |
| Young maternal age (≤20 y) | 1.30 (0.76, 2.21) | 0.336 |  |  |
| Illiterate (cannot read) | 0.90 (0.53, 1.52) | 0.698 |  |  |
| Smoking | 1.45 (0.71, 2.95) | 0.303 |  |  |
| Primigravida (Primipara) | 1.77 (1.07, 2.92) | 0.025 | 1.39 (0.76, 2.54) | 0.278 |
| Overweight | 0.90 (0.50, 1.64) | 0.738 |  |  |
| Pre-eclampsia or eclampsia | 1.97 (0.48, 0.08) | 0.344 |  |  |
| Haemoglobinopathies | 2.08 (1.05, 4.09) | 0.035 | 1.54 (1.06, 2.24) | 0.024 |
| Obstetric characteristics |  |  |  |  |
| Rupture of membranes ≥ 18h | 2.33 (1.10, 4.93) | 0.026 | 4.14 (1.88, 9.10) | <0.001 |
| Oxytocin infusion | 0.84 (0.34, 2.09) | 0.704 |  |  |
| Delayed cord clamping | 1.00 (0.46, 2.19) | 1.000 |  |  |
| Neonatal Characteristics |  |  |  |  |
| Resuscitation | 0.95 (0.23, 3.90) | 0.944 |  |  |
| Presence of haematoma | 2.17 (0.79, 5.99) | 0.133 | 2.15 (0.74, 6.27) | 0.161 |
| Sgaw Karen ethnicity | 1.87 (1.01, 3.46) | 0.046 | 1.67 (0.68 4.08) | 0.260 |
| Male sex | 1.44 (0.87, 2.38) | 0.159 |  |  |
| Small for gestational age | 1.39 (0.77, 2.45) | 0.257 |  |  |
| Sibling with history of jaundice | 1.02 (0.48, 2.16) | 0.952 |  |  |
| Use of naphthalene for storing the clothes | 0.60 (0.15, 2.45) | 0.474 |  |  |
| G6PD deficiency (by FST) | 2.83 (1.35, 5.94) | 0.006 |  |  |
| Potential ABO incompatibility | 0.96 (0.46, 2.02) | 0.917 |  |  |
| Positive Coombs test | 0.49 (0.07, 3.52) | 0.477 |  |  |
| Clinical events |  |  |  |  |
| Severe infection 0-24h | 1.35 (0.42, 4.30) | 0.616 |  |  |
| Weight loss ≥7% at 24h [12-30h] of life | 1.27 (0.31, 5.22) | 0.736 |  |  |
| HCT at 24h [12-30h] of life | 0.86 (0.60, 1.24) | 0.420 |  |  |
| Polycythaemia (HCT >70%) at 24 [12-30h] of life | 0.86 (0.31, 2.37) | 0.769 |  |  |

*WT: wild type; HR: Hazard ratio; CI: confidence interval.*

*^a^Adjusted for Primigravida Rupture of membrane ≥ 18h, Presence of hematoma, Sgaw Karen ethnicity and genotyping of G6PD, UGT1A1*6 and UGT1A1 promoter with p<0.15 from univariate model. G6PD deficiency by FST were* *significant in univariable model but not be included in the multivariable model because they were highly correlated with G6PD genotyping.*
