# Supplementary material for: Contribution of genetic factors to high rates of neonatal hyperbilirubinaemia on the Thailand-Myanmar border
Source: PLOS Glob Public Health. 2022 Jun 17;2(6):e0000475. doi: 10.1371/journal.pgph.0000475 (PMC10021142; doi:10.1371/journal.pgph.0000475)
Supplement: S7 Table — (DOCX) [file pgph.0000475.s007.docx]

**Contribution of genetic factors to high rates of neonatal hyperbilirubinaemia on the Thailand-Myanmar border**

**S7 Table**. Median total serum bilirubin concentrations over time by genotype

|  | Median total serum bilirubin level (IQR) μmol/L | | | |
| --- | --- | --- | --- | --- |
|  | 24h | 48h | 72h | 168h |
| **G6PD genotype** |  |  |  |  |
| Wide-type | (N=897)  112 (98, 128) | (N=615)  172 (145, 199) | (N=502)  202 (165, 237) | (N=568)  201 (152, 246) |
| Hemi-homo/heterozygote | (N=186)  124 (110, 141) | (N=119)  194 (168, 232) | (N=90)  222 (186, 254) | (N=86)  211 (160, 268) |
| Heterozygote | (N=109)  122 (109, 136) | (N=71)  194 (169, 229) | (N=57)  222 (187, 248) | (N=54)  188 (154, 247) |
| Hemi-homozygote | (N=77)  130 (116, 149) | (N=48)  195 (165, 235) | (N=33)  223 (181, 258) | (N=32)  248 (203, 289) |
|  |  |  |  |  |
| **UGT1A1*6 genotype** |  |  |  |  |
| Non-homozygote | (N=1,048)  114 (100, 131) | (N=710)  175 (147, 203) | (N=573)  204 (168, 238) | (N=639)  200 (152, 246) |
| Homozygote | (N=34)  119 (108, 137) | (N=23)  199 (170, 218) | (N=18)  256 (220, 282) | (N=14)  294 (262, 347) |
|  |  |  |  |  |
| **G6PD x UGT1A1*6 genotype** |  |  |  |  |
| ***G6PD: Wide-type*** |  |  |  |  |
| UGT1A1*6: non-homozygote | (N=873)  112 (98, 128) | (N=600)  171 (144, 199) | (N=490)  201 (164, 233) | (N=557)  200 (152, 244) |
| UGT1A1*6: homozygote | (N=24)  116 (103, 135) | (N=15)  185 (170, 214) | (N=12)  265 (236, 289) | (N=11)  312 (274, 354) |
|  |  |  |  |  |
| ***G6PD: hemi-homo/heterozygote*** |  |  |  |  |
| UGT1A1*6: non-homozygote | (N=175)  124 (111, 141) | (N=110)  194 (167, 232) | (N=83)  222 (185, 254) | (N=82)  206 (159, 267) |
| UGT1A1*6: homozygote | (N=10)  135 (110, 152) | (N=8)  213 (174, 232) | (N=6)  225 (186, 258) | (N=3)  262 (251, 313) |
|  |  |  |  |  |
| ***G6PD: hemi-homozygote*** |  |  |  |  |
| UGT1A1*6: non-homozygote | (N=72)  130 (116, 149) | (N=44)  195 (161, 235) | (N=30)  222 (175, 254) | (N=30)  241 (196, 276) |
| UGT1A1*6: homozygote | (N=4)  143 (126, 174) | (N=3)  221 (204, 240) | (N=2)  239 (220, 258) | (N=1)  313 (313, 313) |
