# Supplementary material for: Contribution of genetic factors to high rates of neonatal hyperbilirubinaemia on the Thailand-Myanmar border
Source: PLOS Glob Public Health. 2022 Jun 17;2(6):e0000475. doi: 10.1371/journal.pgph.0000475 (PMC10021142; doi:10.1371/journal.pgph.0000475)
Supplement: S8 Table — (DOCX) [file pgph.0000475.s008.docx]

**Contribution of genetic factors to high rates of neonatal hyperbilirubinaemia on the Thailand-Myanmar border**

**S8 Table.** Proportion (%) of newborns with prolonged jaundice at each follow-up week according to genotype

|  |  | Week 2 | | Week 3 | | 1 Month | |
| --- | --- | --- | --- | --- | --- | --- | --- |
|  |  | N | % | N | % | N | % |
| G6PD | WT | 1127 | 37.3 | 1033 | 32.7 | 1196 | 14.0 |
|  | Heterozygote | 134 | 35.1 | 131 | 33.6 | 144 | 16.0 |
|  | Homo/hemizygote | 100 | 50.0 | 95 | 44.2 | 102 | 25.5 |
|  |  |  |  |  |  |  |  |
| UGT1A1*6 | WT | 935 | 34.8 | 860 | 29.5 | 985 | 12.2 |
|  | Heterozygote | 385 | 43.1 | 360 | 40.6 | 411 | 17.8 |
|  | Homozygote | 40 | 62.5 | 38 | 60.5 | 45 | 51.1 |
|  |  |  |  |  |  |  |  |
| UGT1A1*28 | WT | 855 | 39.1 | 807 | 33.3 | 916 | 15.2 |
|  | Heterozygote | 222 | 42.8 | 210 | 40.5 | 225 | 20.4 |
|  | Homozygote | 26 | 50.0 | 26 | 38.5 | 24 | 29.2 |
|  |  |  |  |  |  |  |  |
