# Supplementary material for: Contribution of genetic factors to high rates of neonatal hyperbilirubinaemia on the Thailand-Myanmar border
Source: PLOS Glob Public Health. 2022 Jun 17;2(6):e0000475. doi: 10.1371/journal.pgph.0000475 (PMC10021142; doi:10.1371/journal.pgph.0000475)
Supplement: S9 Table — (DOCX) [file pgph.0000475.s009.docx]

**Contribution of genetic factors to high rates of neonatal hyperbilirubinaemia on the Thailand-Myanmar border**

**S9 Table**. Haematocrits tested at 24 hours in neonates born from mothers with different haemoglobin types.

| Haemoglobin typing  (mother) | N | Mean Hct (neonate) | SD |
| --- | --- | --- | --- |
| Hb Normal | 848 | 59.0 | 7.2 |
| Beta-thalassaemia trait | 64 | 59.4 | 8.6 |
| Beta-thalassaemia/Hb E disease | 2 | 61.0 | 0.1 |
| Hb E trait | 51 | 59.0 | 6.9 |
| Hb Hope trait | 1 | 56.0 | - |
| Homozygous HbE | 2 | 55.0 | 1.4 |
