# Supplementary material for: Contribution of genetic factors to high rates of neonatal hyperbilirubinaemia on the Thailand-Myanmar border
Source: PLOS Glob Public Health. 2022 Jun 17;2(6):e0000475. doi: 10.1371/journal.pgph.0000475 (PMC10021142; doi:10.1371/journal.pgph.0000475)
Supplement: Attachment — Submitted filename: NH_PGPH_rev_responses_all.docx [file pgph.0000475.s010.docx]

We would like to thank the reviewers for suggesting improvements to the manuscript.

Here below, our point-by-point responses to the comments raised by the reviewers.

Reviewer #1: Reviewer comment
Line 34-35: Increased red blood cell turn-over and immaturity of hepatic glucuronidation both contribute. Revise this statement in terms of language structure

*This has been re-phrased and it now reads: “Both increased red blood cell turn-over and immaturity of hepatic glucuronidation contribute to increased levels of bilirubin”*

Line 36: define ca

*This has been change to “approximately”*

Line 46: state specific haemoglobinopathies

*We have re-worded as “maternal mutations on beta-globin gene”*

Line 49: define EGA
*This has been defined*

Lines 38, 43: state specific mutations identified to be associated with NH
*We have re-phrased to: “G6PD mutations and UGT1A1*6 mutation, together with….”*

Line 70: for reader comprehension, define Viangchan (871G>). This is because the resulting amino acid was not indicated

*The typo has been corrected to “Viangchan (871G>A).*

Lines 54-94: most references are outdated. Provide current references and current information
*We politely disagree on this with the reviewer as several references provided are recent (<5years). Many of the older references are about the basic biology of G6PD and UGT1A1 mutations. Recent references are discussed more in depth in the discussion section.*

Line 135, there was an open bracket but not a close bracket. Insert it appropriately

*We have inserted the close bracket.*

Line 224-225: it is not clear why authors excluded twins from the study. explanation to this decision is expected

*For the calculation of allelic frequencies, only unrelated subjects should be included. For the analysis of risk, twins are usually born smaller and earlier and therefore at increased risk of NH independently from their genetic background or other clinical risks factors. Furthermore, twins represented only roughly 1% of the entire sample. We therefore have decided to exclude twins from the analysis. We have modified the sentence, it now reads: “a small percentage (1.2%, N=20) were twins and were excluded from the genetic analysis because related. Twins were also excluded from the risk analysis because they are often born smaller and earlier independently from their genetic background or other clinical factors.”*

Line 285: Tables 2-6, define WT

*This has been added at the bottom of the Tables.*

Reviewer #2: Thank you for the opportunity to review this paper. Congratulations to the team and also apology for delay in reviewing.

Several comments/suggestion:
- Though the title is self explanatory, in the abstract it would be beneficial to add one line stating the aim of the study, and also in method to mention the study design

*A sentence was added in the abstract that reads: “We aimed to investigate the contribution of genetic risk factors to high levels of bilirubin in the first month of life in 1596 neonates in a prospective observational birth cohort study”.*

- In method: please specify the phototherapy used - because in many Asian communities, the tradition to sunbath newborn is quite common. How does the cultural factors are taken into account into the study procedures?

*Indeed, sunbath is common and a practice observed in the clinic while the newborn is in the postnatal ward and as long as SBR levels remain below the PT threshold. Nevertheless, the phototherapy units were set up in the clinics in 2019 and they have been well accepted by the mothers.*

*We have added the following sentences to the methods:*

*“Two types of bulbs for phototherapy were available: Phillips TL20/52 blue light bulbs* *of 400 to 500 nm wavelength and LED bulbs (peak wavelength 455 nm). The blue-light bulbs were either inserted into a wooden or a metallic framed cot; LED bulbs units were mobile and set directly above the baby cot. PT units delivered recommended minimal irradiance levels of 8-10 µW/cm^2^/nm; the distance between the light and the cot was adjustable in order to reach, if necessary, intensive phototherapy (≥30 μW/cm^2^/nm, Hyperbilirubinemia 2004). The first phototherapy units were set up in the clinics in 2009 (Turner 2013) and by the time of this study were well accepted by the mothers who could sleep near the cot, breastfeed, and care for their newborn.”*

- Please, specify why clustering was important to be taken into account in each model.

*Accounting for clustering was important because members of the same cluster (site) would tend to have more correlated outcomes in general compared to members of a different cluster (site). Failure to account for these correlations would often tends to bias p-values downwards thereby increasing type I error. We have included a sentence in the manuscript to explain this in the methods section.*

- Results: very thorough and detailed, use of table is good to visualize what otherwise lost in the dense narrative. The use of PAR as well is very appropriate to express the relative contribution of the studies variables. What were the main assumptions used and why the mixed effect binomial model was used for the prolonged icterus.

*The main assumption when using PAR was that the* ***sample that was used to estimate the prevalence of the risk factor in the population at large was a random sample of the population. The outcome was binary, hence, the most appropriate model was the mixed effect binomial model as it accommodates the modelling of the binary outcome.*** *The mixed effect part of the model was important because we assumed that the intercept for each individual site is random (random intercepts) meaning that each site represents some other sites in the population that have the same characteristics as the site that is included in the model, hence, the mixed effects model captures the expected site variation in the outcome in the population. Random effects model is statistically robust for analysing clustered data.*

- Discussion; The limitations mentioned only other genetic factors that were not investigated in this study, is it because the study was conducted back in 2016-17?

*Based on literature review and budget availability, we have analysed the two major genetic factors associated to NH. Should research funding become available in future, we might be able to investigate other genetic factors listed in the discussion using biobanked samples from the study.*

- What are the recommendations that applies to this setting? Would it be feasible and desirable to As said in line 539 on availability of sequencing, do the authors refer to high income countries?

*Cheap sequencing is becoming increasingly available in middle income countries (but not low) but without a proper cost analysis it is difficult to predict exactly in which context genotyping of the mother (or both parents or newborn) might represent a cost-effective approach. In this specific setting, where we do not have high-throughput cheap genotyping available, collection of maternal and perinatal risk factors, G6PD phenotypic testing at birth together with close follow-up of newborns remain the best available option (and indeed the one used in SMRU clinics).*

- The conclusion also mentions diagnosis of G6PD, but availability of the desired product (quantitative) seems to be sub optimal, is there any development in the pipeline worth mentioning?

*There are currently at least 3 different quantitative or semi-quantitative point-of-care G6PD tests in late stage of development and one that we have recently evaluated specifically in newborns. We have added this sentence to the discussion: “One such test has been recently evaluated with good results in this setting among newborns (Bancone, in preparation) and few more are in the late stage of development”*
